# Neutron Diffraction and Diffraction Contrast Imaging for Mapping the TRIP Effect under Load Path Change

**DOI:** 10.3390/ma13061450

**Published:** 2020-03-23

**Authors:** Efthymios Polatidis, Manuel Morgano, Florencia Malamud, Michael Bacak, Tobias Panzner, Helena Van Swygenhoven, Markus Strobl

**Affiliations:** 1 Laboratory for Neutron Scattering and Imaging, Paul Scherrer Institute, Villigen PSI, CH-5232 Villigen, Switzerland; michael.bacak@psi.ch (M.B.); tobias.panzner@swissneutronics.ch (T.P.); 2 CONICET, Laboratorio Argentino de Haces de Neutrones, Departamento de Física de Neutrones, Centro Atómico Bariloche, San Carlos de Bariloche R8402AGP, Argentina; fmalamud@cab.cnea.gov.ar; 3 Swissneutronics AG, CH-5313 Klingnau, Switzerland; 4 Swiss Light Source, Paul Scherrer Institute, Villigen PSI, CH-5232 Villigen, Switzerland; helena.vanswygenhoven@psi.ch; 5 Neutrons and X-rays for Mechanics of Materials, IMX, Ecole Polytechnique Federale de Lausanne, CH-1015 Lausanne, Switzerland

**Keywords:** TRIP, load path change, neutron, Bragg edge

## Abstract

The transformation induced plasticity (TRIP) effect is investigated during a load path change using a cruciform sample. The transformation properties are followed by in-situ neutron diffraction derived from the central area of the cruciform sample. Additionally, the spatial distribution of the TRIP effect triggered by stress concentrations is visualized using neutron Bragg edge imaging including, e.g., weak positions of the cruciform geometry. The results demonstrate that neutron diffraction contrast imaging offers the possibility to capture the TRIP effect in objects with complex geometries under complex stress states.

## 1. Introduction

The preferred deformation mechanisms in austenitic stainless steels strongly depend on the stacking fault energy (SFE) [1,2,3]. When the SFE is sufficiently low, the transformation induced plasticity (TRIP) effect occurs upon deformation [4]. Several studies have focused on the investigation of the TRIP effect in this class of materials under different monotonic load paths. A few examples are uniaxial tensile [5,6,7,8], compression [9] loading or complex states such as bending [10], torsion [11], and biaxial loading [12,13,14].

Sheet metals and alloys are often subjected to biaxial loadings and load path changes (LPCs) during their forming processes. The mechanical behavior of TRIP steels under LPCs is poorly understood. In addition, the existing transformation kinetic models fail to predict the TRIP effect evolution upon LPCs as they typically only account for the cumulative strain upon monotonic loading. With the development of multiaxial loading rigs [15,16,17,18], monotonic and LPCs can be applied using cruciform-shaped samples. Cruciform samples have the advantage of being able to be used for proportional or non-proportional loading paths. Typically, the center of the cruciform is investigated by either X-ray [16,19,20,21] or neutron [22,23] diffraction or microscopy [12,20,24]. In addition, the stress state at the center of the cruciform can be computed with the aid of Finite Element (FE) simulations [25]. While high strains need to be reached in the center of the cruciform, where characterization using diffraction methods are applied, FE simulations predict that stress concentrations and complex stress states appear at the cross-arms leading usually to failure upon deformation [25].

The biaxial test rig installed at the POLDI (Pulse-OverLap DIffractometer) beamline [15,26] at the Swiss spallation neutron source (SINQ), at the Paul Scherrer Institute, Switzerland has been employed for studying the twinning induced plasticity in stainless steel 316 (SS316) [22], the transformation induced plasticity in SS304 [14] and SS201 [13], and LPCs on SS316 [27]. For instance, it was shown that the TRIP effect can be suppressed or facilitated during monotonic loading, phenomena that can be explained by the interplay of loading state, the crystallographic texture, and SFE [13,14]. In particular, the medium SFE SS304 steel exhibits deformation twinning under uniaxial loading which suppresses the transformation of the parent face-centered cubic (fcc) phase into body-centered cubic (bcc) martensitic phase. Twinning appears in the majority of the grains because they are favorably oriented for the formation of extended stacking faults under uniaxial tension which is a precursor for twinning formation [28]. In contrast, deformation twinning is suppressed under biaxial loading in the majority of the grains, and hence the martensitic transformation dominates the plastic deformation [14]. The deformation behavior of this material under LPC has however not yet been studied. It is however to be expected that the TRIP effect will depend on the multiaxial deformation history. One of the major difficulties is the fact that designing cruciform geometries for such studies is a rather difficult task, and usually the optimal geometry is material dependent [25]. It is well known that complex stress states appear locally in cruciform geometries, and these can interfere with the LPC. Usually, FE simulations are performed to predict the localized stress states [29]; validation of these predictions is usually missing.

Diffraction of X-rays or neutrons provide information related to the crystal structure of the materials, such as lattice strain, phase, crystallographic texture, micro strain, and crystallite size averaged over a limited gauge volume. In traditional diffraction experiments, Bragg peaks are observed at wavelengths for which the Bragg equation is fulfilled. The diffraction patterns are recorded as a function of diffraction angle for monochromatic instruments at continuous sources [30], or as a function of wavelength and diffraction angle at time-of-flight instruments, mainly at pulsed sources [31]. In transmission-based diffraction contrast imaging, also referred to as Bragg edge imaging, scattering information is extracted from wavelength resolved transmission data [32]. Here the diffracted neutrons are missing in the transmission signal, thus leading to an increased attenuation at wavelengths subject to significant Bragg scattering. Bragg edges appear in transmission spectra of powder-like polycrystalline samples at wavelengths twice the lattice spacing, *d_hkl_*, beyond which no Bragg diffraction from the specific *hkl* lattice plane family can take place anymore, and thus the transmission increases significantly. These Bragg edge features in the transmission spectra probed by wavelength resolved neutron imaging enable mapping and local analyses of crystalline phases and strains in 2D [33] and 3D [34,35]. As such, neutron diffraction contrast imaging can complement conventional neutron diffraction, and due to its superior spatial resolution it is ideal for investigating complex sample geometries, phase distributions and corresponding lattice strain fields [32,34,35,36,37,38,39,40].

Here, we investigate the TRIP effect under a 90-degree LPC performed on a cruciform SS304 sample. The transformation at the center of the cruciform was followed during an in-situ neutron diffraction experiment at the POLDI beamline at SINQ. The sample was then mapped using neutron diffraction contrast imaging, allowing the visualization of the spatial distribution of transformed regions in the cruciform. This experiment was carried out using a tunable double crystal monochromator at the BOA beamline at SINQ [41]. The neutron diffraction contrast imaging reveals the presence of martensite at locations of stress concentrations, which were found to agree well with predictions by FE simulations.

## 2. Materials and Methods 

A commercial AISI304 (EN 1.4301) stainless steel in sheet form of 8 mm thickness was purchased from SAUTER EDELSTAHL AG, Switzerland. The as-received material exhibits relatively random crystallographic texture and an average grain size (excluding the annealing twins) of 35 μm; more details about the initial microstructure and crystallographic texture are given in [14]. Cruciform-shaped samples with the geometry proposed in [3,4] were used for the equibiaxial tests. The cruciform geometry features a gradual thickness reduction from 8 mm at the gripping parts down to 2 mm at the center of the cruciform. A uniform thickness of 2 mm is machined within a circular section of 24 mm in diameter.

**Figure 1:**
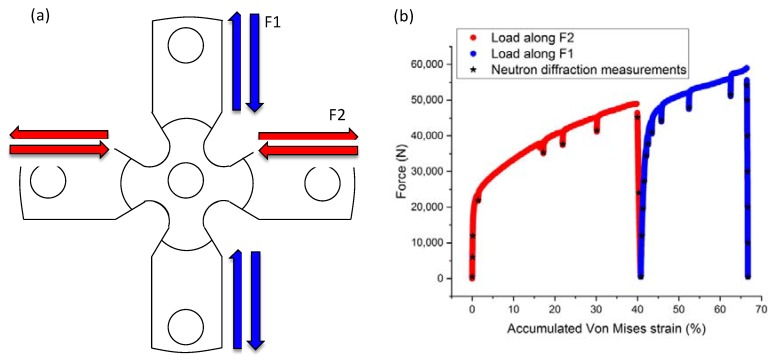
(**a**) Schematic representation of the cruciform geometry and the 90 degrees load path change, i.e., loading/unloading F2 followed by loading/unloading F1. (**b**) Force-accumulated Von Mises strain plot during the load path change showing the two load paths, loading along F2 (red) and along F1 (blue). The strain values at which neutron diffraction measurements were undertaken are shown with black asterisks.

The material was deformed using the biaxial deformation rig of the POLDI beamline at the Swiss spallation neutron source, SINQ [5]. The cruciform sample was initially deformed along the rolling direction (direction F2) in load control, with a loading rate of 80 N/s up to a maximum force of 50 kN. The sample was then unloaded to 0 N, followed by deformation along the F1 direction with a loading rate of 80 N/s, up to a maximum force of 60 kN. The neutron diffraction measurements were carried out in predefined force intervals after interrupting the loading and holding the displacement (see Figure 1b) using a gauge volume of 3.8 × 3.8 × 2 mm^3^ gauge volume. The biaxial deformation system is equipped with a 2-camera digital image correlation (DIC) system (GOM Aramis 5M, GOM GmbH, Braunschweig, Germany) for measuring the in-plane strain at the center of the cruciform sample. The DIC strain was averaged over an area of 3.8 × 3.8 mm^2^, which corresponds to the area illuminated by the neutron beam during the neutron diffraction experiments. During holding the displacement, force/stress relaxation is recorded due to creep, as seen in the characteristic dips in Figure 1b. The maximum equivalent strain reached at the center of the cruciform during each of the load paths is approximately 41% and 26%, respectively. The accumulated Von Mises strain for both load paths is, hence, approximately 67%.

The energy resolved neutron imaging measurements for phase content mapping were performed at the test beamline BOA (Beamline for neutron Optics and other Approaches) [41] of the spallation neutron source, SINQ, at the Paul Scherrer Institute. This instrument is particularly well-suited for Bragg Edge imaging due to the cold, relatively intense and clean spectrum available, which is the result of a neutron supermirror bender which efficiently transports a large fraction of cold neutrons, while the instrument is not directly viewing the source, thus avoiding fast neutron and gamma background. This combination enables a good signal-to-noise ratio crucial for long exposure times implied by wavelength resolved measurements. Wavelength selection was achieved by a double crystal monochromator (DCM) [42] employing Bragg reflection by two large (100 × 40 mm^2^) pyrolytic graphite crystals to select neutron wavelengths in the range from 2.4 Å to 6 Å, with a full width at half maximum (FWHM) of about 0.05 Å, and providing a sufficiently large and homogenous beam for imaging in forward direction.

An IKON-M CCD camera (Andor-Oxford Instruments, Abingdon, UK) coupled with a 100 mm lens was used as a detector for these measurements, achieving a field of view (FoV) of approximately 60 × 60 mm^2^ and a pixel size of 60 µm. The neutrons were converted to light with a 200 µm thick 6LiF-based scintillator. Together with the neutron aperture used (40 × 40 mm^2^ at 6 m distance for an L/D of 150), the detection resulted in a spatial resolution of ~200 µm. To achieve a sufficient signal to noise ratio in each image, an exposure time of 25 min was used.

For the measurements reported here, we collected 51 equidistant images in the range between 2.5 Å and 4.5 Å with a step size of 0.04 Å. This resulted in a total exposure time of ~21 h for each sample and the same for the corresponding open-beam imaging. Several dark images with 25 min exposure time were also acquired for offset correction.

The acquired wavelength resolved images were processed with conventional outlier removal filters, offset correction, detector background correction, and flat field normalization. The values obtained by taking the natural logarithm of the normalized images are directly proportional to the total macroscopic cross section of the material at the corresponding energy and the thickness of the material. Normalizing the results with the known local material thickness provides the local wavelength dependent cross section for each pixel of the transmission detector.

Bragg diffraction is dominating the cross section in the respective wavelength range, displaying in particular the fcc (111) and (200) Bragg edges, as well as the bcc (110) and (200) Bragg edges for mixed γ-austenite and α’-martensite phases respectively. Thus, the respective phase fractions can be mapped through such experimental approach [35,36].

**Figure 2:**
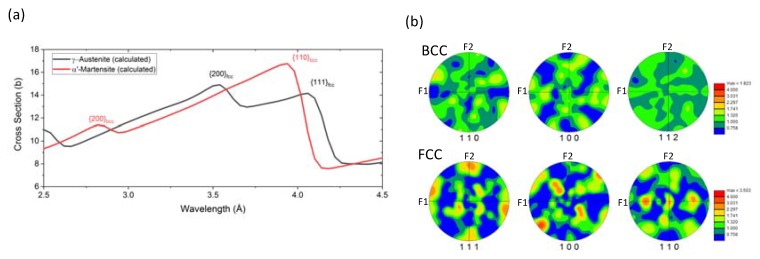
(**a**) Theoretical attenuation spectra for γ-austenite and α’-martensite incorporating the corrections for absorption due to alloying, crystallographic texture, and instrumental effects. (**b**) Pole figures depicting the crystallographic texture at the center of the cruciform that were used for correcting the theoretical attenuation spectra to account for the crystallographic texture effect.

**Figure 3:**
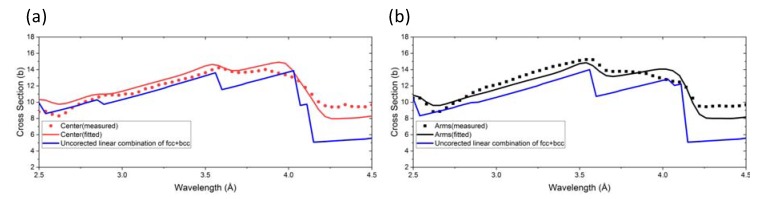
Measured (points), refined uncorrected (blue), and refined corrected (red or black lines) Bragg edge curves for two locations, (**a**) center of the cruciform with 60% γ-austenite (fcc) and 40% α’-martensite (bcc), and (**b**) arms of the cruciform with 91% γ-austenite (fcc) and 9% α’-martensite (bcc).

Figure 2a shows the calculated attenuation spectra for 100% γ-austenite and 100% α’-martensite. These spectra have been calculated accounting for (1) the alloying elements of SS304, (2) the effect of crystallographic texture, using the pole figures (Figure 2b measured at the center of the sample with electron backscatter diffraction (EBSD) and applying the correction in [43,44], (3) the second order diffraction that affects the selection of wavelengths from the DCM, and (4) its wavelength resolution dλ/λ = 0.05 Å (FWHM). The measured spectra were then least-square fitted using a linear combination of the calculated spectra of γ-austenite and α’-martensite (Figure 3).

Figure 3 exemplarily shows the refined curves, using the corrected attenuation spectra, with respect to the experimental data obtained at two locations of the sample, i.e., the center of the cruciform (Figure 3a) and at the arms of the cruciform (Figure 3b). For comparison, the Bragg edge curves using a linear combination of bcc and fcc spectra, without incorporating any corrections, i.e., assuming pure Fe, random crystallographic texture, and ignoring the instrumental effect on the attenuation spectra, are shown as blue lines. The uncorrected spectra for pure iron were calculated using the open software nxsPlotter [45]. It is evident that the applied corrections improve the agreement between the experimental data and the modelled curves significantly, especially the smoothening effect of the strong crystallographic texture and instrumental effects on the Bragg edges (compare the blue and red curves in Figure 3a and the blue and black curves in Figure 3b). It should be noted that the crystallographic texture, due to deformation, is expected to vary significantly within the cruciform sample due to spatially varying stress states and stress localizations. Here we have applied a correction based on the crystallographic information obtained at the center of the cruciform, where the deformation is expected to be high. Nevertheless, the correction based on the crystallographic texture at the center of the cruciform gives good agreement between the modelled and the experimental data also at the arms of the cruciform. For the 2D phase maps, the fitting procedure was undertaken pixel-wise with running average of 3 pixels × 3 pixels.

## 3. Results

**Figure 4:**
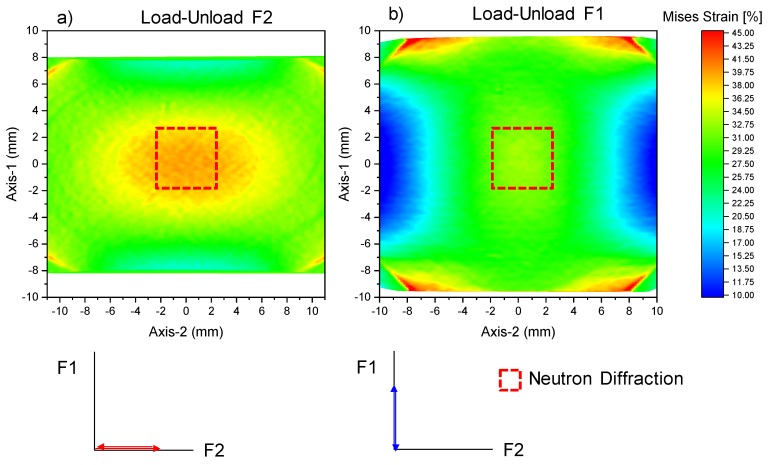
Digital image correlation (DIC) map showing the strain after load and unload along (**a**) F2. (**b**) DIC map of the strain field generated from the subsequent load-unload along F1. In both load paths, strain concentrations are seen at the circular part of the cruciform.

Figure 4 shows the strain distribution in the field of view of the DIC system at POLDI at the end of each load path. For the calculation of these maps, a new reference image is used at the beginning of the corresponding load path, and hence the maps shown in Figure 4 represent the strain reached at the end of each load path only. DIC captures the relatively uniform strain distribution at the center of the cruciform, where the neutron diffraction investigation is undertaken. Aside from uniform strain at the center of the cruciform, the DIC reveals strain concentrations at the boundaries of the circular thickness reduction (cf. Figure 4).

**Figure 5:**
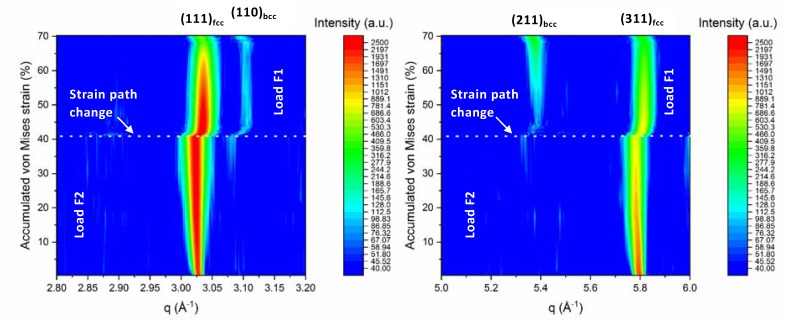
Neutron diffraction evolution from in-situ deformation along the 90-degree load path change, separated along two ranges of q, i.e., 2.8 Å to 3.2 Å (left) and 5 Å to 6 Å (right). The diffraction patterns show the evolution of the (111)_fcc_ and (311)_fcc_ austenite and the appearance of (110)_bcc_ and (211)_bcc_ martensite reflections upon the change of the load path.

**Figure 6:**
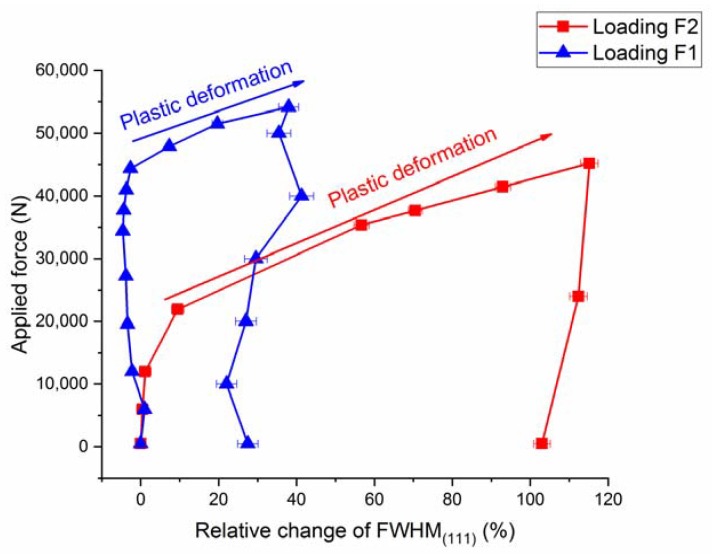
Evolution of the full width at half maximum (FWHM) of the (111)_fcc_ reflection for the two load paths showing pronounced peak broadening due to dislocation-based plasticity along path 1 (i.e., loading F2), less peak broadening appears in load path 2 (i.e., loading F1).

As mentioned in the introduction, the TRIP effect is suppressed under uniaxial loading for SS304, as seen in Figure 5, where the neutron diffraction pattern does not exhibit pronounced α’-martensite peaks upon the first load path, i.e., loading along F2. A weak martensite reflection appears at strain >35% during the first loading path. As a result of the suppressed TRIP effect under uniaxial loading (loading F2), the deformation is dominated by dislocation slip which is seen as pronounced peak broadening due to an increase of the density of dislocations and slip in Figure 6. In contrast, upon changing the load path (i.e., loading F1), the deformation is dominated by the martensitic phase transformation, as shown in Figure 5, where strong martensite reflections appear in the neutron diffraction pattern. In contrast to the first load path, the increase of FWHM is not as pronounced during the second load path, as shown in Figure 6. It should be noted that the transformation and dislocations activity is dependent on the crystallographic orientation, with respect to the loading direction [9,13,14,46]. In Figure 6, the normal to the {111} planes is parallel to the loading direction, F2, and the neutron scattering vector, q. The <111>-oriented grains can be divided into sub-sets of grains according to their crystallographic alignment with respect to second loading axis (loading along F1). Therefore, the observed broadening of the (111) reflection can be affected by the alignment of each grain with respect to the loading direction F2.

**Figure 7:**
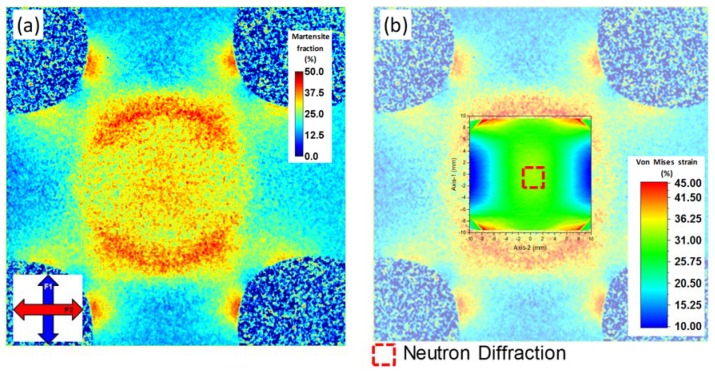
(**a**) Neutron diffraction contrast imaging showing the distribution of martensite within the cruciform sample. (**b**) The localization of martensite agrees well with the strain localization as obtained by digital image correlation.

Figure 7a shows the diffraction contrast imaging phase map, which clearly depicts deformation-induced martensite concentrations. It is seen that martensite preferentially forms at locations where stresses typically concentrate during deformation of cruciform samples. Moreover, the center of the cruciform sample, which is mapped with neutron diffraction, exhibits a relatively uniform distribution of martensite with an average fraction of approximately 40%, which agrees very well with the averaged martensite fraction of approximately 41% obtained by EBSD. Interestingly, some small fraction of martensite (<10%) is seen in the images to be present at the arms of the cruciform, where the deformation is expected to be elastic, and thus no martensitic transformation is taking place. A simple test using a magnet, however, showed that the undeformed material is magnetic, which can only be explained by the presence of low volume fractions (<10%) of delta ferrite [47], confirming the Bragg edge imaging results, implying a residual bcc phase fraction. This observation thus underlines that diffraction contrast imaging is especially sensitive in capturing even relatively small fractions of phases. Overlaying the DIC strain map of the second load path (i.e., loading along F1) with the diffraction contrast image of the martensite distribution shows a good matching of the in-plane strain concentrations and locations of high fractions of strain-induced martensite (Figure 7b).

## 4. Discussion and Prospects of the Method

The 90-degree LPC results in a significant amount of strain-induced martensite, higher than what is observed during equibiaxial loading in [14]. During the first load path, martensite does not form while in the plastic regime; significant slip activity and increase of the density of dislocations take place. Typically, the slip systems with the highest Schmid factor are activated, as it has been observed using high resolution DIC using scanning electron microscopy [6,24,48]. As a result of high plastic deformation from the load path 1 (loading F2), the yield point of the subsequent loading (loading F1) increases significantly. Upon the LPC and as the loading direction is rotated at 90 degrees with respect to the first load path, new slip systems are activated. In similarity with the high resolution DIC observations on monotonic loadings [6,24,48], the new activated slip systems, during the subsequent load path, have the highest Schmid factor. The activation of multiple shear systems and the intercepts of shear bands create ideal conditions for martensite nucleation [4,24,49,50], therefore a 90-degree LPC results in pronounced TRIP effect in SS304.

**Figure 8:**
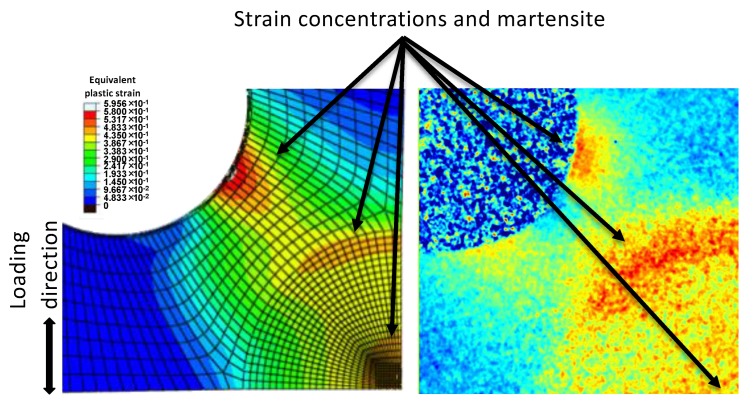
Finite element simulation under uniaxial loading (**left**) showing the strain localizations which correlate well with the concentration of strain-induced martensite, as captured by neutron diffraction contrast imaging (**right**).

Neutron diffraction contrast imaging is capable of capturing very well the spatial distribution of martensite induced due to stress concentrations, as presented in Figure 8. A FE simulation was carried out using the commercial ABAQUS/Standard software, (Version 6.14, Dassault Systèmes, Providence, RI, USA). The stress-strain curve and isotropic elastic properties of SS304 obtained from uniaxial loading of a dogbone sample are used as materials properties input to the built-in model based on the Von Mises yield criterion and the associated flow rule. In order to improve the computational efficiency, only 1/8th of the entire cruciform geometry was simulated with symmetric boundary conditions being applied on the appropriate surfaces. A structured hexahedron mesh was used with linear 8-node C3D8. The simulation was undertaken with a linear loading (surface traction mode) to a target force of 50 kN along only one direction. The subsequent loading was not simulated due to lack of mechanical properties of the material with prior deformation. Nevertheless, neutron diffraction contrast imaging captures well the locations that FE modeling predicts strain localization under uniaxial loading. Moreover, this method opens new potentials for spatially-resolved simulations and applications of kinetic models. By combining 2-dimensional maps of strain-induced martensite fractions, obtained from diffraction contrast imaging, with the stress state variations obtained from FE simulations and the equivalent strain magnitudes obtained from DIC, it is possible to apply kinetic models that can describe the martensite phase fraction as a function of strain magnitude [4,51,52] and/or stress state [53,54,55]. Applications where diffraction contrast imaging for phase/strain analysis has already been undertaken are torsion tests [34], bending tests where the strain (and in association to strain the strain-induced martensite) varies from tensile, through a neutral line to compression [56], and complex stress states in fatigue samples. Potential new applications could include bulge tests that the strain and stress states vary significantly from place to place [57], deformation tests on metallic foams [58,59], and additively manufactured open cell structures [60,61,62] that exhibit local complex stress states and strain concentrations.

## 5. Conclusions

A cruciform sample of austenitic stainless steel was subjected to uniaxial load path change. The TRIP effect is suppressed upon the initial uniaxial loading; however, upon loading the second direction, a significant amount of martensite appears. It is suggested that this results from the activation of secondary slip systems during the subsequent load path, which intercept with the slip systems that are activated during the first load path. Based on the established nucleation theory for deformation-induced martensite, the intercepts of multiple shear bands are preferred locations for martensite formation. Neutron diffraction contrast imaging was undertaken using a double crystal monochromator and collecting neutron transmission images in the range between 2.5 Å and 4.5 Å with a step size of 0.04 Å. The Bragg edge curves were in good agreement with the modelled curves, when appropriate corrections were applied to account for the crystallographic texture, cross sections of the alloying elements and instrumental effects. The results, however, also underline that texture plays an important role in phase quantification and that simple approaches unable to account for texture risk significant bias in respective results [34]. Otherwise, the method is demonstrated to be capable of capturing the TRIP effect with high spatial resolution within a field of view of several cm^2^. Neutron diffraction contrast imaging confirms the uniform distribution of strain-induced martensite at the center of the cruciform, where the neutron diffraction investigations were undertaken. In addition, strain-induced martensite is seen to form at locations where DIC captures strain concentrations and/or FE simulations predict stress concentrations such as the circular section at the center of the cruciform and at the cross-arms. Neutron diffraction contrast imaging is a powerful method that can be utilized for in-situ investigations using complex sample geometries under complex stress states.
